# Diagnostic Feature Extraction and Filtering Criterion for Fatigue Crack Growth Using High Frequency Parametrical Analysis

**DOI:** 10.3390/s21155030

**Published:** 2021-07-24

**Authors:** Ángela Angulo, Cristinel Mares, Tat-Hean Gan

**Affiliations:** 1 TWI Ltd., Granta Park, Great Abington, Cambridge, Cambridgeshire CB21 6AL, UK; tat-hean.gan@twi.co.uk; 2 College of Engineering, Design and Physical Sciences, Brunel University London, Kingston Lane, Uxbridge, Middlesex UB8 3PH, UK; Cristinel.Mares@brunel.ac.uk

**Keywords:** parametrical analysis, signal feature extraction, high-frequency signals, filtering criterion, fatigue crack growth, mooring chains

## Abstract

Mooring systems are an integral and sophisticated component of offshore assets and are subject to harsh conditions and cyclic loading. The early detection and characterisation of fatigue crack growth remain a crucial challenge. The scope of the present work was to establish filtering and alarm criteria for different crack growth stages by evaluating the recorded signals and their features. The analysis and definition of parametrical limits, and the correlation of their characteristics with the crack, helped to identify approaches to discriminate between noise, initiation, and growth-related signals. Based on these, a filtering criterion was established, to support the identification of the different growth stages and noise with the aim to provide early warnings of potential damage.

## 1. Introduction

Mooring systems are an integral and complex component of offshore assets. Mooring chains for floating offshore installations, typically designed for a 25 year service life, are subject to fatigue in a seawater environment. Structural components are subjected to harsh conditions and cyclic loading and can present a significant risk to asset integrity and personnel safety. Moreover, the life of old and new structures has to be extended, and the maintenance strategies improved, calling for a more proactive management approach.

Structural health monitoring (SHM) is a crucial part of asset integrity management and should be enforced in conjunction with traditional inspection programmes. The main objective of SHM is to continuously verify the condition of the mooring system’s performance and provide input for the assessment of mooring integrity, complementing periodical in-service inspections. The early detection and monitoring of fatigue crack growth in mooring chains remain a crucial challenge. Current technologies cannot provide information on fatigue cracking, ultimately leading to unexpected structural failure. Thus, its detection and characterisation open the way towards a new, reliable monitoring approach that can be used as an early warning of crack propagation.

One of the major problems in the design of offshore equipment is fatigue damage accumulation, predominantly at the chain connection at the fairlead where failure can occur rapidly [1]. Although this topic has been extensively studied in the literature, theoretically, numerically, and experimentally [2,3,4], the available inspection and monitoring technologies developed to date have not been able to fully overcome the severe environmental challenges associated with offshore service activities. ROVs have been widely used since the 1970s but face serious difficulties, despite the technological advances, due to the highly unpredictable operating environment characterized by poor visibility and unstable conditions [5,6,7]. For fatigue damage detection in structural applications in general, several sensing techniques have been developed [8] including guided ultrasonic waves [9,10], fibre Bragg gratings (FBGs) [11,12,13], strain gauges [14], and piezoelectric sensors [15].

Amongst the offshore assets that are vulnerable to corrosion-enhanced fatigue damage, mooring chains are one of the most crucial mooring components used in permanently anchored structures [16]. Despite their importance, limited experimental work on mooring chain fatigue cracking monitoring exists in the literature. Studies have been carried out on the chains’ material microstructural properties [17], whereas others have been numerical modelling-oriented [18]. Few large-scale testing attempts have been made [19], conducting feasibility studies to establish acoustic techniques’ capabilities in monitoring damage in a mooring chain link subject to stress corrosion cracking in artificial sea water.

The present research presents a diagnostic feature extraction for vibration sensor-based diagnosis technologies and evaluates the suitability of monitoring parameters of high-frequency signals to detect fatigue crack growth in mooring chains. Experimental work was performed to investigate the applicability of the technique and the accuracy of waveforms’ feature extraction to monitor and predict fatigue-related cracking in mooring systems. 

This investigation results in the definition of a data filtering criterion for the prediction and assessment of fatigue crack growth in mooring chains, characterising four different clusters of events, three of which are directly related to the cracking phases: initiation, growth, and final growth. Following these results, a colour-coded alarm for risk identification can provide warnings based on low, medium, high, and very high-risk stages.

## 2. Materials and Methods

The testing was conducted at TWI Ltd.’s world-class mechanical test rig facility. The work generated reliable test data for a more fundamental understanding of the parameters providing information on real-time fatigue cracking. The test was executed in steel grade R5 links of 127 mm in diameter. The rig was designed to test a segment of a chain in a seawater environment, including seven studless links, and the rig allowed a chain section to be tested horizontally under cyclic tension–tension loading. All links were fully immersed in the solution during the duration of the fatigue and the testing.

### 2.1. Fatigue Testing Set Up and Results

The chain was subjected to a constant cycling tensile loading (tension–tension). During the 31 days of the monitoring period, an average tension–tension load range of 3475 kN to 3755 kN was applied.

A displacement limit was set during the fatigue testing, measured by a high-sensitivity displacement cell. This limit could be exceeded due to the potential development of a crack, or in some cases, other operational circumstances. The aim was also to find an early indication of fatigue cracking. If, after the trigger of the displacement cell, it was considered that the source could be a potential crack developing in one of the links, the full tank would be emptied, and consecutive NDT would be carried out from end to end of the complete chain section. 

**Figure 1:**
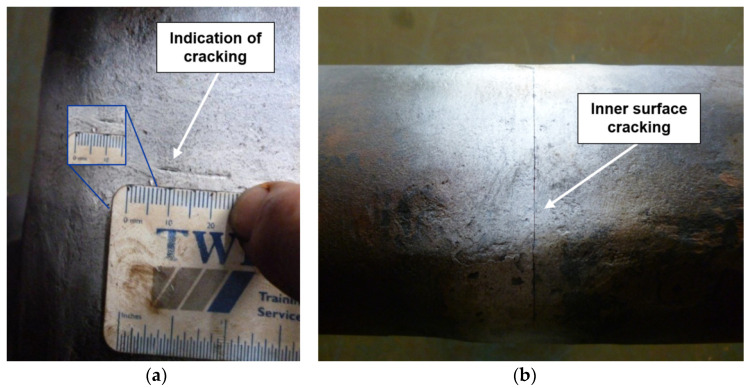
Crack indications after magnetic particle inspection (MPI): original crack found at the weld on the inner surface of link #4, (**a**) 5 mm long to (**b**) 120 mm long.

The targeted fatigue life for the tested sample was 5 × 10^6^ cycles, as established by the manufacturer’s original calculations. From the deployment of the monitoring equipment at 2,656,544 cycles, the first time the displacement limit was exceeded occurred after 4,333,424 cycles. Subsequently, the water tank was fully drained and a complete inspection of the chain using visual inspection and magnetic particle inspection (MPI) revealed a ~5 mm crack located at the weld area in link #4 (Figure 1a). No other indication of cracking was found thus the tank was re-filled and the experiment was resumed. 

At 4,923,552 cycles, close to the fatigue life (5 × 10^6^ cycles), the experiment was interrupted for a second time. MPI revealed that the same crack that was originally found in link #4 (4,333,424 cycles, 5 mm) had grown to ~120 mm (Figure 1b). A complete inspection of the chain did not reveal any other cracks in the other links.

Fatigue failures are often easy to identify. The initiation region usually occurs on the surface. The 5 mm crack initiated in link #4 was originated with a smooth surface and then developed into a rougher area as the crack increased in size, following the crack growth trajectory. This behaviour is characteristic of the fatigue crack propagation phase. A region corresponding to the final 120 mm fatigue crack displayed a granular grey portion outside the crack’s arc, corresponding to the crack final fracture, and was artificially opened at low temperature by applying a critical load to reveal the fracture surfaces.

### 2.2. The Effect of Cyclic Loading in Fatigue Crack Growth

Structural components subjected to cyclic loading can fail even if the stress level remains well below the failure stress observed under static or monotonic loading. This mechanical failure mechanism is what defines fatigue damage.

Previous research has investigated fatigue crack growth in relation to the applied load. Cracks could grow during the loading and the unloading phase of the load cycle [20]. Nevertheless, any correlation attempt may be unsuccessful when the complete loading range is considered. Some studies have attributed the top 10% and 5% of the load range to crack growth-related [21] events. Other research has associated different signal groups with the crack behaviour based on a spectral analysis of the data [22], concluding that the signals generated at the top 75–85% of the load range were statistically dissimilar from those in the bottom 60% range and were associated with the fatigue crack growth.

The effect of cycling loading in the present research work was analysed and it was confirmed that activity related to crack initiation and growth always occurs during a cycle ascending phase and at 85% of the top load range. In addition, those events occurring at loads over 97% can be considered an indication of crack growth present in the sample. These values will initiate the filtering criteria to be determined and analysed through consecutive sections.

## 3. Monitoring Approach

Depending on the application and the monitoring strategy, two different approaches are generally applied to acquire and analyse the vibration signals [23].

- Continuous type: refers to a waveform where transient bursts are not discriminated against. This is the most common type of signal processing for the analysis of vibration in rotating machinery.
- Burst type emission: burst type emission is expected when the monitored sources of emission are non-repeatable and occur discretely; independent in the time domain, such as local catastrophic yielding, crack growth, cavitation, or corrosion.

When the origin of these waves travelling through the material is due to plastic deformation, as atomic planes slip past each other through the movement of dislocations, the fundamental characteristic of these signals is that they are in a broad frequency range between 100 kHz and 1 MHz. In this case, these types of high-frequency vibration are generally known as acoustic emissions (AEs). AE is defined as a rapid release of strain energy caused by deformation or damage within or on the surface of a material that generates transient elastic waves.

Approaches in logging and analysing AE data can be separated into two main groups: parameter-based (classical) and signal-based techniques [24]. Other analysis approaches of transient signals such as spectral, phase space, and wavelet methods can also be utilised to process acoustic signals [25]. Techniques for the evaluation transient signals such as spectral, phase space, and wavelet methods can also be utilised to process AE data. More modern techniques such as artificial neural networks (ANNs) can be applied to analyse and cluster signals and parametrical data [26]. These are, however, not within the scope of the present work.

**Figure 2:**
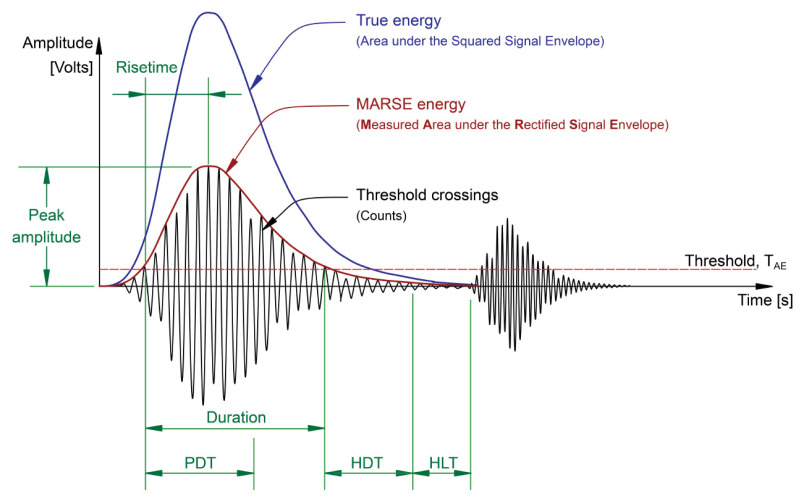
Illustration of the threshold-based hit detection and features extracted from each hit [27].

The most common technique to define burst type emissions is based on the signal threshold. The threshold is usually set on the positive side of the signal and fixed just over the average noise level. It can be adjusted regularly if the noise levels change in the environment. Once a burst has been located, it must be determined. There are typically three parameters that are used for this: the hit definition time (HDT), the hit lockout time (HLT), and the peak definition time (PDT). Figure 2 illustrates the threshold-based hit detection method and the main features under discussion.

The HDT states the maximum time between threshold crossings, meaning that if no signal crossing occurs during this time, then the hit has ended. Practically, if the HDT is set too high, the system may consider two or more hits as one. If the HDT is set too low, the system may not fully capture the burst and possibly treat one hit as multiple hits. The HLT parameter specifies the time which must pass after a hit has been detected before a new hit can be detected. In this case, if the HLT is set too high, then the system may not capture the next event, and if it is set too low, then the system may capture reflections and the late arriving component of the hits. The PDT parameter specifies the time allowed after a hit has been detected to determine the peak value. If the PDT is set too high, then false peak value measurements are more likely to occur [28,29]. 

The determination of the burst, also known as the hit, is the first step before extracting the hit-based features. The most common and predominantly used parameters are amplitude, duration, energy, counts, rise time, and average frequency. Figure 2 illustrates how these and other common hit-based features are related [30], and each of the key signal features is described below [27,28,29,30,31,32,33]:

- Amplitude (A-dB): the greatest measured voltage in a waveform is measured in decibels (dB). Amplitudes are expressed on a decibel scale instead of a linear scale where 1µV at the sensor is defined as 0 dB. The amplitude is closely related to the magnitude of the source event, and signals with amplitudes below the minimum threshold will not be recorded. In general, the value does not represent the emission source but the sensor response after losing the energy due to propagation.
- Duration (D-µs): the time interval between the first and last threshold crossings. The duration is generally expressed in microseconds, which depends on source magnitude and noise filtering. Like counts, duration relies upon the magnitude of the signal and the acoustics of the material.
- Count (C) (also known as ring-down count/emission count): the number of times within the duration when one signal exceeds a pre-set threshold. Counts depend strongly on the employed threshold and the operating frequency. While this is a relatively simple parameter to collect, it usually needs to be combined with amplitude and duration measurements to provide quality information about the shape of a signal.
- Rise time (RT-µs): the time interval between the first threshold crossing of the signal and the time of its peak amplitude. The rise time is closely related to the source-time function and is commonly used to qualify signals or a noise filter criterion.
- Average frequency (AF-kHz): a calculated feature obtained from counts divided by duration, determining an average frequency over one hit.

### 3.1. Equipment

**Figure 3:**
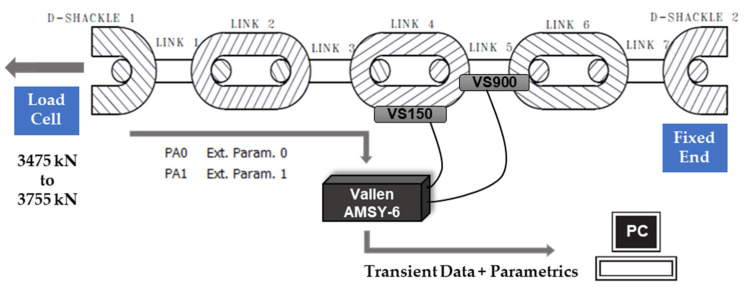
Parametric inputs (PA0 load and PA1 displacement) and attributes calculated from transient data from sensors at link #4 and link #5.

During the duration of the monitoring period, two different sensors with resonant and bandwidth frequencies were placed in links #4 and #5 of the mooring chain (see Figure 3).

Data were collected using a data acquisition Vallen AMSY-6, a fully digital multi-channel measurement system, and sensors.

- Link #4: VS150-WIC-V01 (resonant frequency 150 kHz, bandwidth 100–450 kHz);
- Links #5: VS900-WIC-V01 (resonant frequency 350 kHz, bandwidth 100–900 kHz).

### 3.2. Calibration

The sensors used in this study were calibrated following the ASTM E1106 [34] standard before starting the test to ensure the consistency of the collected signals. Since the experiments were to be completed with the chain submerged in water, the reproducibility of the response of the sensor was verified in air and underwater according to ASTM E976 [35] by breaking a 0.5 mm pencil lead against the link’s surface, known as the pencil lead breakage (PLB) test. Also known as Hsu and Nielsen [36] pencil lead break, PLB is a recognised technique used to artificially generate reproducible signals [37]. PLBs were performed in groups of four, at +10 cm and −10 cm from each sensor. Additionally, to verify sensor coupling, the pulsing function was applied. Each sensor was used as a signal generator that transmits signals to be captured by the rest of the sensors. Details of the calibration plan are summarised in Table 1.

### 3.3. Data Acquisition Parameters 

Setting up the acquisition parameters is critical for optimum performance. The acquisition sampling rate was set at 10 MHz. The transient data were recorded at 5 MHz, taking 200 samples for the pre-trigger and 200 post duration. For the definition of the hit, the duration was set at 400 µs, the rearm time at 3200 µs, and the hit threshold at 60 dB. The frequency range was left at 95–850 kHz for a broader frequency perspective. The data acquisition threshold value was set at 60 dB due to the noisy environment. 

Trend analysis of hit-based features is widely used when dealing with high-frequency signals, e.g., in AE, and so burst signal features, such as the time of the first threshold crossing (time of arrival), duration, peak amplitude, energy, or counts, were extracted from the ASIP-2 (AMSY-6 signal processor) using AE-Suite software. External parametric inputs were recorded for the load (PA0) and displacement (PA1).

### 3.4. Data Collection Procedure 

During the 31 days of the monitoring period, an average tension–tension load range of 3475 kN to 3755 kN was applied. The crack of interest was revealed 17 days before the end of the experiment. The periods leading to both the 5 mm indent and the 120 mm crack were analysed and compared with the activity during normal rig operation before the occurrence of any cracking.

Data were collected in windows with a duration of 3 to 8 days. Throughout the processed monitoring period, data were split into four parts. This was due to two main reasons. Firstly, the test rig natural load and unpredicted triggers (e.g., invalid system triggers or cracks revealed at the end of period 2) also caused time loss between the acquisition periods. Second, the size or duration of the files collecting transient data (e.g., file size/time for period 1 over 9 GB and period 3 over nine days). This also helped to keep the files to a practical and similar size to perform the post-processing. The details and characteristics of each one of them are described in Table 2.

The nomenclature used in this table will be followed through the following sections and figures when describing the data results, namely: P1, P2, P3, P4. It is important to remember that P2 corresponds to the crack initiation stage, leading to C1, initial 5 mm crack observed, and P3 and P4 correspond to the crack growth phase, leading to C2, the discovery of the 120 mm crack, and the end of the experiment.

## 4. Results

### 4.1. A Qualitative Overview of the Cumulative Energy per Period 

The energy of a burst signal is defined as the integral of the squared signal (voltage signal) divided by the reference resistance, over the duration of the waveform. The qualitative reading of a cumulative energy graph should take into account the following existing conditions. First, if the slope is zero, no events are occurring. Second, if the slope is relatively constant and the individual energy measured per event is low, the energy is most often coming from environmental noise. However, if there is a manifestation of a sudden rise in the cumulative energy indicated by the increase in the slope, this can be an indication of the presence of an anomaly. 

**Figure 4:**
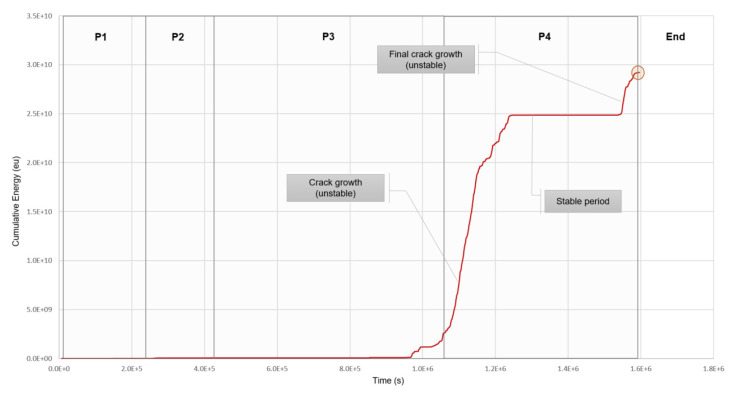
Cumulative energy (eu) vs. time (s) during the full duration of the monitoring period (P1, P2, P3, and P4) for sensor #4. Data filter based on load > 3700 kN and amplitude over 80 dB applied.

Figure 4 shows the cumulative energy versus time for the duration of the experiment when the events with loads under 3700 kN and amplitudes under 80 dB were discarded. 

### 4.2. Evaluation of Burst Parameters and Their Relationship to Define a Filtering Criterion

Parametrical analysis of signals’ features is crucial for an evaluation and classification of characteristic bursts concerning the type of damage and/or stage occurring at a given time interval. These can support assessing and filtering the representative features’ values for different cracking stages (initiation, growth, and final growth). The definition of these limits will result in the establishment of a filtering criterion, which will ultimately be used to provide a qualitative evaluation of the risk associated with the findings.

#### 4.2.1. Peak Amplitude vs. Signal Energy 

**Figure 5:**
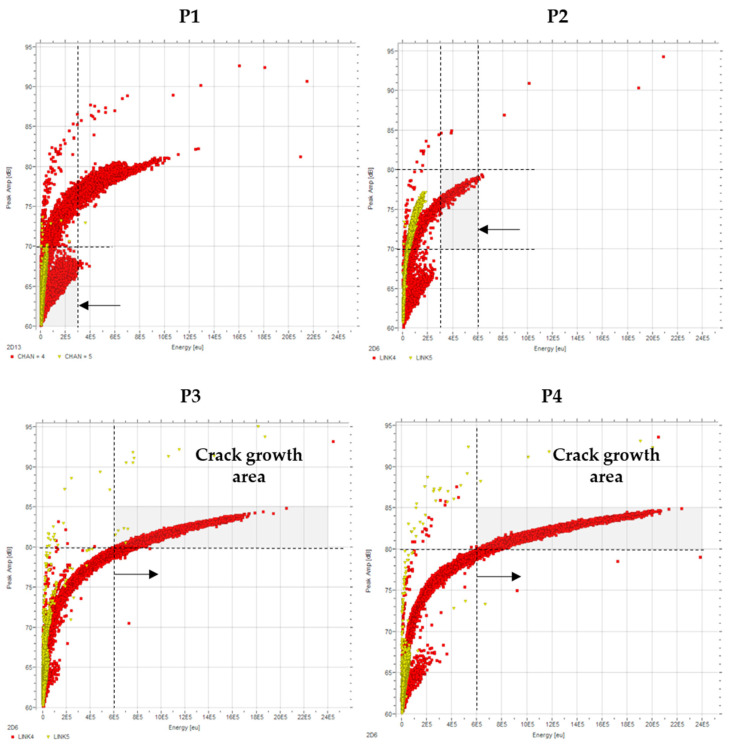
Peak amplitude (dB) vs. energy (eu) evolution for the four periods based on raw data.

High-energy and high-amplitude signals are generated when crack initiation or unstable crack extension occurs [38]. Figure 5 shows the evolution of the amplitude versus energy for all periods. P1 and P2 display a slow rise in energy as the amplitude increases. Only a few events go over the 80 dB limit and 6 × 10^5^ eu. However, looking at P3 and P4, it can be observed how the energy increases very quickly as the amplitude increases. There is a change in the curve slope, where a small amplitude increase (from 80 dB to 85 dB) results in a high energy values increase (from 6 × 10^5^ eu to 22 × 10^5^ eu). This is an indication of the presence of relatively high-frequency signals, containing a large number of counts per hit while still maintaining a reasonably high duration, and it is characteristic of signals linked to cracking [39].

These results indicate unstable crack growth-related activity in P3 and P4, given the small increase in amplitude, resulting in a quick increase in energy per hit. 

#### 4.2.2. Counts vs. Peak Amplitude 

Signal counts have also been used during fatigue crack propagation tests, and the results from the previous research [6] agree that as the crack growth rate increases, the count rate increases. 

The energy of a signal can be expressed by an area of a triangle that consists of the width of signal duration and the height of peak amplitude. Therefore, if the energy of a burst increased according to the crack growth, the peak amplitude would increase as well as the count rate. 

**Figure 6:**
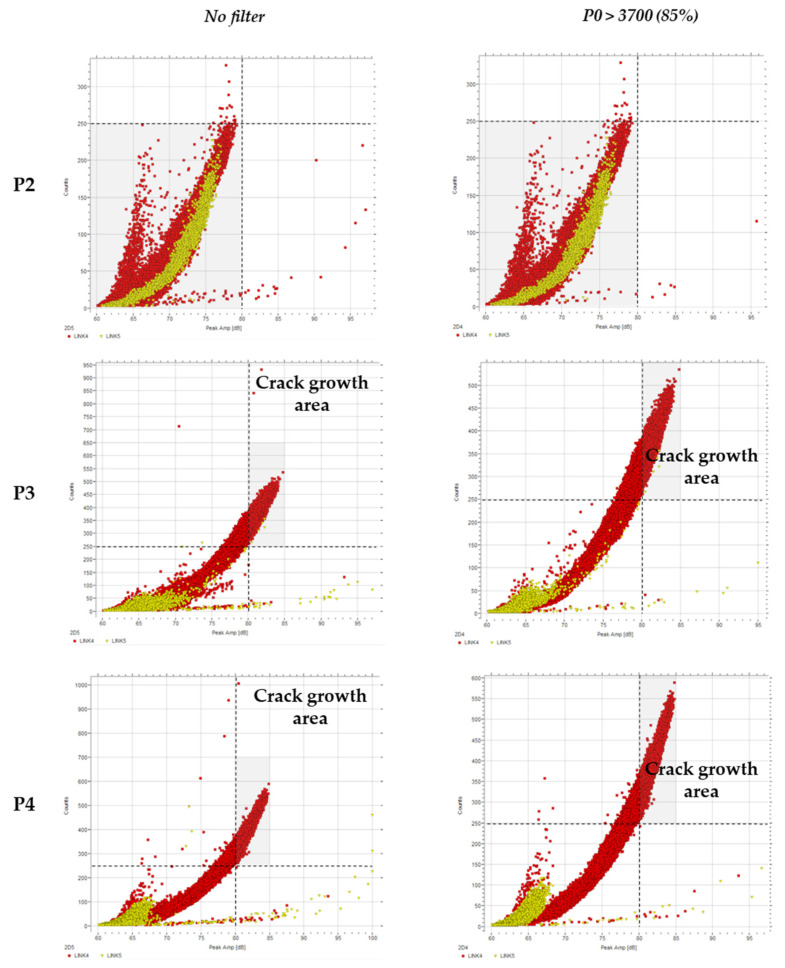
Counts (c) vs. amplitude (dB) filtered by the load.

Discarding the signals recorded under 85% of the top load range, a plot of the burst counts versus amplitude was created for the three damage stages: initiation, growth, and ultimate growth (Figure 6). The number of counts quickly increases with the increase in amplitude (from 80 to 85 dB) to over 250 counts per hit.

When entering the plastic stage, the count rate gradually increases, indicating that cracks begin to propagate [39]. Therefore, this result is in agreement with the sudden increase in energy caused by a small increase in amplitude as described in Section 4.2. The more counts the event contains, the more energy the signal will carry. 

#### 4.2.3. Duration vs. Peak Amplitude 

**Figure 7:**
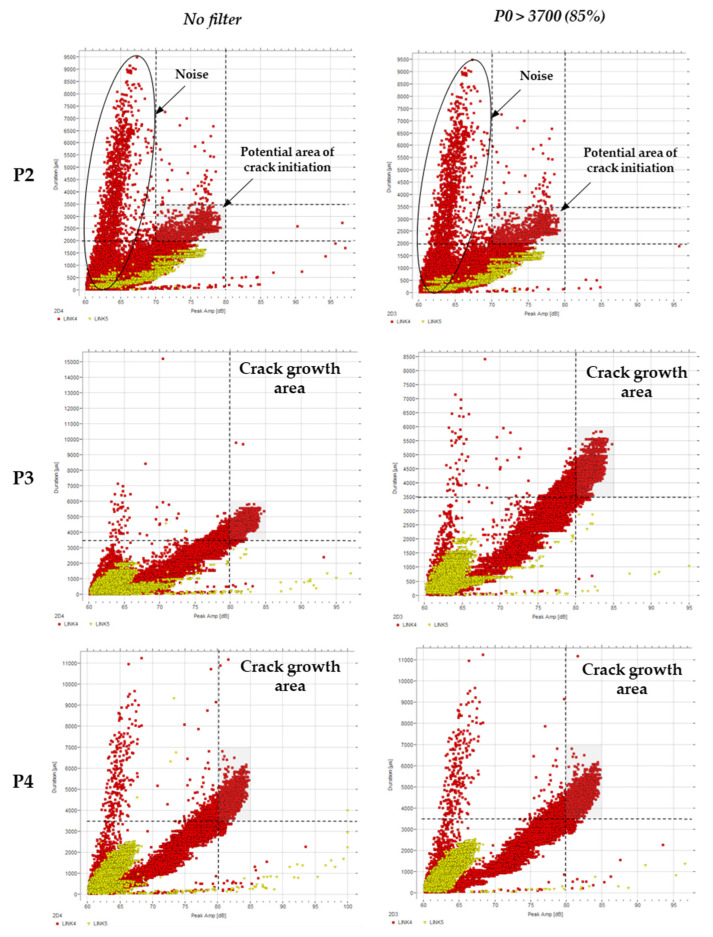
Duration (µs) vs. amplitude (dB) filtered by the load.

The relationship between duration and amplitude for the three data sets (P2, P3, P4) is shown in Figure 7. It can be observed that these were only slightly affected by the fatigue cycle as there was no clear difference in the results according to the loading filtering (over 85% of the top load range). P2, which was defined as the stabilisation to initiation period, displays a number of long-duration events with low amplitudes. These (highlighted with a circle) and signals with a duration under 2000 µs can be considered friction noise and therefore discarded. 

During normal activity, the amplitude rarely reaches values above 80 dB (Figure 7), and the duration appears to fall within 0 to 3500 µs, and it remains higher for larger amplitudes. Duration is the elapsed time from the first threshold crossing to the last. As occurs for energy and counts, as the amplitude increases, the duration of threshold crossing increases. More importantly, the duration increases to values exceeding 6500 µs, two times as large as the maximum duration for ‘normal’ activity. The amplitude also increases to at least 85 dB, which is again larger than the largest values observed during ‘normal’ activity.

Of interest is the activity for which duration exceeds 3500 µs, and if there are a significant number of data points above 80 or 85 dB. It can then be confirmed that these long-duration signals are attributed to events generated from the crack growth stage of the test.

#### 4.2.4. Peak Amplitude vs. Duration vs. Energy

When fracture occurs, the duration increases with amplitude, energy, and number of counts, as described in previous sections. A 3D correlation plot of the signal amplitude, energy, and duration can support the analysis and validation of the entire signals to confirm characteristics, trends, and limits. Previous research [40] has used correlation plots based on this three-dimensional relationship and cited that earlier literature shows crack growth behaviour characterised by short-duration, high-amplitude, and high-energy signals. Conversely, this same research defined the duration of the corresponding crack propagation to be greater than the crack initiation and any fretting. 

**Figure 8:**
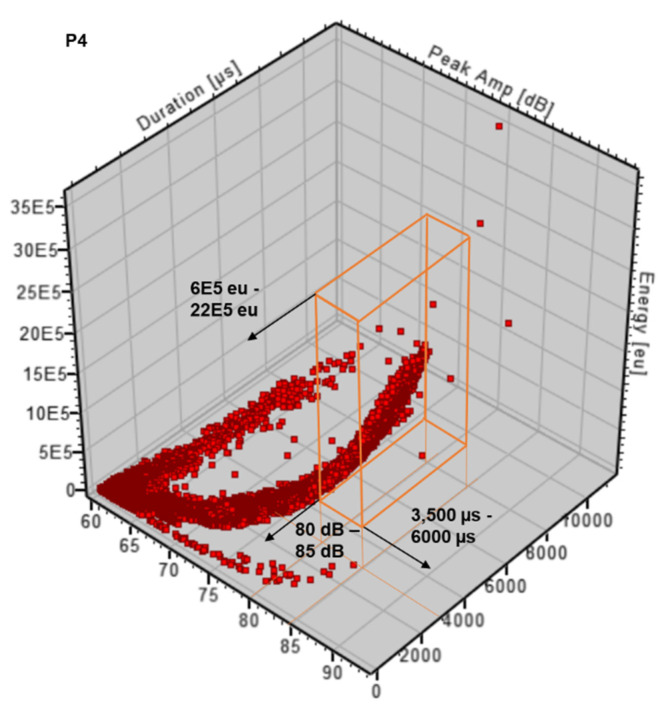
Peak amplitude (dB) vs. duration (µs) vs. energy (eu) (no filter applied).

During the present experiment, the cycling loading tests in P4 showed that the energy increases very quickly as the amplitude increases, especially in signals over 80 dB, where the energy ranges from 6 × 10^5^–22 × 10^5^ eu (see Figure 8). Low-amplitude activity is seen for short-duration events, with energy reaching a maximum of 5 × 10^5^ eu, so this is at least one order of magnitude smaller than that for the crack growth regions. A few hits exceed 85 dB, but none exceed 90 dB, set as the test limit.

Based on the results of Section 4.2 and Figure 7, the 3D correlation plot shown in Figure 8 corroborates the previous outcome for P4 and defines the characteristic signal caused by crack propagation as a long-duration, high-amplitude, and high-energy pulse: 3500–6000 µs, 80–85 dB, and 6 × 10^5^–22 × 10^5^ eu.

#### 4.2.5. Average Frequency: Counts over Duration 

**Figure 9:**
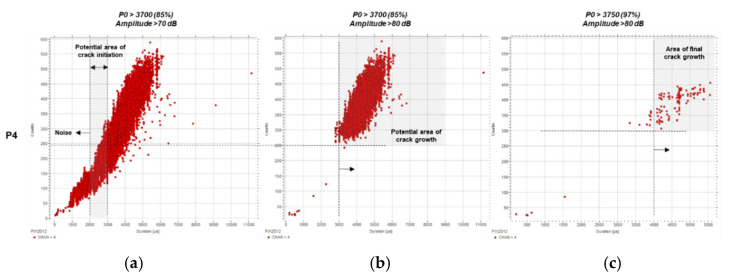
Counts (c) over duration (µs) for P4 using predefined filters: (**a**) *P0* > 3700 (85%) and A > 70 dB, (**b**) *P0* > 3700 (85%) and A > 80 dB, and (**c**) *P0* > 3750 (97%) and A > 80 dB.

**Figure 10:**
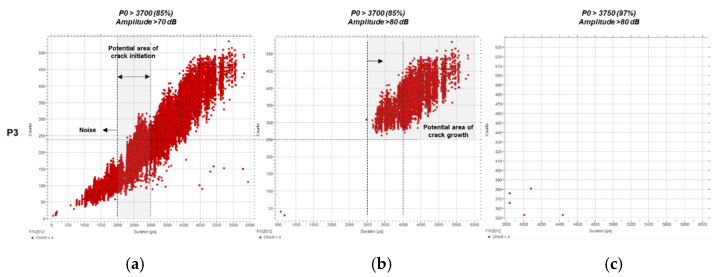
Counts (c) over duration (µs) for P3 using predefined filters: (**a**) *P0* > 3700 (85%) and A > 70 dB, (**b**) *P0* > 3700 (85%) and A > 80 dB, and (**c**) *P0* > 3750 (97%) and A > 80 dB.

**Figure 11:**
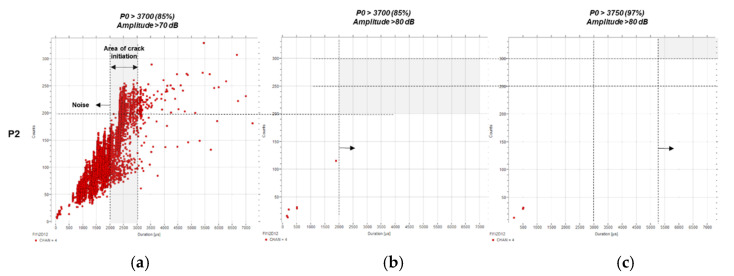
Counts (c) over duration (µs) for P2 using predefined filters: (**a**) *P0* > 3700 (85%) and A > 70 dB, (**b**) *P0* > 3700 (85%) and A > 80 dB, and (**c**) *P0* > 3750 (97%) and A > 80 dB.

**Figure 12:**
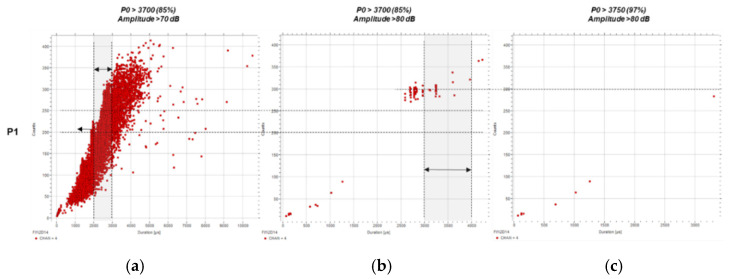
Counts (c) over duration (µs) for P1 using predefined filters: (**a**) *P0* > 3700 (85%) and A > 70 dB, (**b**) *P0* > 3700 (85%) and A > 80 dB, and (**c**) *P0* > 3750 (97%) and A > 80 dB.

Average frequency is defined as the ratio of counts to duration in a burst. An analysis of average frequency and its evolution across the four testing periods has been performed, and the results are shown in Figure 9 (P4), Figure 10 (P3), Figure 11 (P2), and Figure 12 (P1). Data are displayed starting from P4 as the thresholds become conclusive after applying the established load and amplitude filters. 

The first outcome of this frequency analysis is shown in Figure 9, which displays the relationship between counts and duration for P4. The results are plotted for three different filters to support the refinement of the thresholds. Once the second filter was applied (b, *P0* > 3700 and A > 80 dB), the majority of the low-count, short-duration signals were eliminated. The characteristics of the remaining ones correspond to the crack growth phase, with values over 3000 µs and 250 counts. 

As the load filter was raised to 97% of the load, a limited number of hits remained, presumably caused by the final stage cracking. These events are characterised by long durations and a large number of counts, over 4000 µs and 300, respectively.

Revising the results shown in Figure 7 and Figure 8, and looking at Figure 9, the original estimated threshold for crack initiation characterisation should be lowered from 3500 µs to 3000 µs to ensure all potential relevant events are considered. In addition, the characterisation of noise under 2000 µs must be taken into account while setting the limits between the initiation phase and the noise, as indicated in Figure 9. In agreement with the findings displayed on Figure 6, signals over 250 counts should be considered potential events related to crack growth. 

Figure 10 shows the analysis of the counts over duration for P3, still displaying crack growth-related points. The major difference with P4 (Figure 10c) is that there are no hits over the 4000 µs mark. Still, the crack growth area is limited to a low range by events over 250 counts and 3000 µs.

Building on these results, and as anticipated, the ratio for P2, corresponding to the crack initiation phase, shows no data when the filters with amplitude over 80 dB are applied (Figure 11b,c). This result agrees with the filtering criteria defined in previous sections, defining the crack initiation threshold above 2000 µs and under 250 counts. 

Finally, the results obtained for P1 (Figure 12) follow a similar trend but are inconclusive given the large amount of noise present in the file.

In conclusion, it can be established that based on the data files and the filtering criteria, events with a duration above 2000 µs would correspond to crack initiation, those above 3000 µs to growth, and those over 4000 µs to final crack growth. The number of counts would be over 200 for crack initiation, 250 for the crack growth stage, and over 300 for the ultimate cracking. 

The threshold for crack growth definition set at 3000 µs overwrites the limit defined in Figure 7 and Figure 8 (3500 µs) as it is more conservative. 

#### 4.2.6. Average Frequency vs. Number of Hits

**Figure 13:**
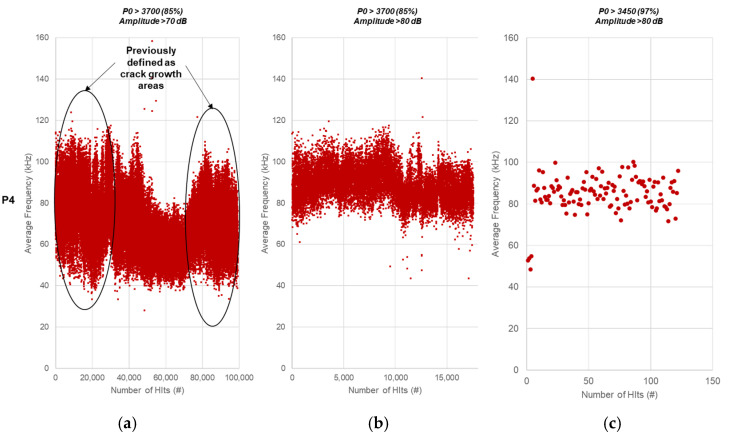
Average frequency over number of hits for P4 using predefined filters: (**a**) *P0* > 3700 (85%) and A > 70 dB, (**b**) *P0* > 3700 (85%) and A > 80 dB, and (**c**) *P0* > 3750 (97%) and A > 80 dB.

Figure 13 shows the average frequency (kHz) over time for each hit recorded using three different predefined filters. The two regions of high amplitude and high energy shown in Figure 13 (for P4) can also be observed to follow a higher-frequency spectrum, as seen in Figure 4. The unfiltered noise signals are expected to have a lower average frequency content, and a large number of those observed when applying the second filter (b) still exhibit low average frequencies, just over the 70 kHz umbral (Figure 13a).

When the high load filter is applied (*P0* > 3750 (97%) and A > 80 dB), only the final crack growth-related hits remain (Figure 13c). These plots provide a trend, where the average frequency of the dataset filtered at 97% of the load and over 80 dB displays average frequencies between 75 kHz and 100 kHz, whereas the data with a 70 dB threshold filter, at 85% of the top load range, displays a much broader range of average frequencies between 40 kHz and over 110 kHz. This means that the frequency range can be narrowed for a specific type of flaw, crack growth on this occasion. 

**Figure 14:**
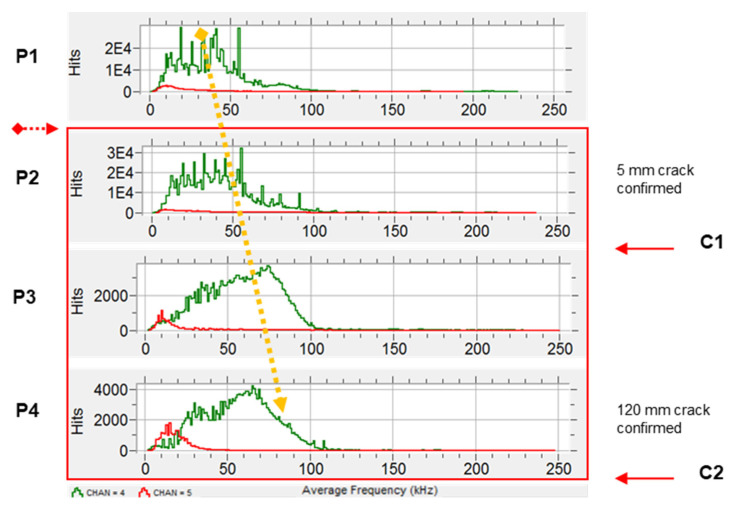
Hits (n) vs. average frequency (kHz). Shift of the average frequency in #4 (green trace).

A broader look at the frequency content of the signals detected during the experiment, presented in Figure 14, shows a continuous shift of the average frequency towards higher values throughout the monitoring period.

The shift in the signal frequency content may be indicative of a change in the damage mode or stage. For example, in composites, the signal frequency can directly correlate to the fracture mechanism [41]. Additionally, it has been found that tensile cracking is associated with a higher signal frequency range when compared to shear cracking in the same class of materials [42].

## 5. Discussion 

### 5.1. Improved Filtering and Integrity Warning Criteria

There were many factors that contributed to the generation of data during the chain fatigue testing. It was demonstrated that each damage stage generated different signal properties, even while producing similar waveforms. The present research has analysed the signals’ typical characteristic parameters (per burst) for identified noise and crack growth stages. Based on this investigation, the compilation of the parametrical filtering criteria for the four major stages is summarised in Table 3. 

The average frequency calculation provides an estimation of the projected and ranged frequency present in crack-related events, but cannot be considered as a filtering criterion.

- Noise is generally characterised by low amplitude, small number of counts, short duration, and low energy. The very early adaptation of the noise filter, if possible at the acquisition stage, will result in a reduced amount of irrelevant data collected and stored. This will be advantageous when trying to process the relevant information, particularly if the intention is to provide real-time figures of the asset’s condition. Therefore, it is recommended that a data recording filter is set at the acquisition stage based on: A < 70 dB, C < 200, D < 2000 µs, and E < 3 × 10^5^ eu. The limitation of duration and number of counts will automatically limit the range of the average frequency range. This simple step would drastically reduce the storage space and processing speed and support more effective data management and display of results.
- At the crack initiation stage, the signals were generated by the local plastic deformation and microcracks when the local surface stress was concentrated. Raising from the characteristic noise phase, events with medium amplitude, moderate number of counts, medium duration, and medium energy were observed for the corresponding signal parameters. It must be noted that the established crack initiation will still include information from signals from noise, friction, and other sources. This means that all the events generated by crack initiation should fall within this range, but not all the events recorded within the set limit will correspond to crack initiation. In summary, the region where the hits, corresponding to crack initiation, will fall within the limits is: 70 dB < A < 80 dB, 200 < C < 250, 2000 µs < D < 3000 µs, and 3 × 10^5^ eu < E < 6 × 10^5^ eu.
- During the crack propagation stage, the events, compared to the previous initiation stage, amplitude, counts, duration, and energy characteristic values increased, and the average frequency was limited to the 70 kHz < AF < 110 kHz range. The characteristic number of counts and duration should be within the 250 < C < 300 and 3000 µs < D < 4000 µs ranges, respectively. Only hits with amplitudes of over 80 dB can be considered to be related to crack growth. Additionally, as the chain entered the unstable crack growth stage, the signal energy quickly rose, showing values of over 6 × 10^5^ eu.
- Events with a large number of counts (C > 300) and long duration (D > 4000) can be directly considered as an indication of a potential presence of an unstable crack growth period when the load exceed 97% of the maximum load applied.

### 5.2. Crack Growth Warning Criteria 

**Figure 15:**
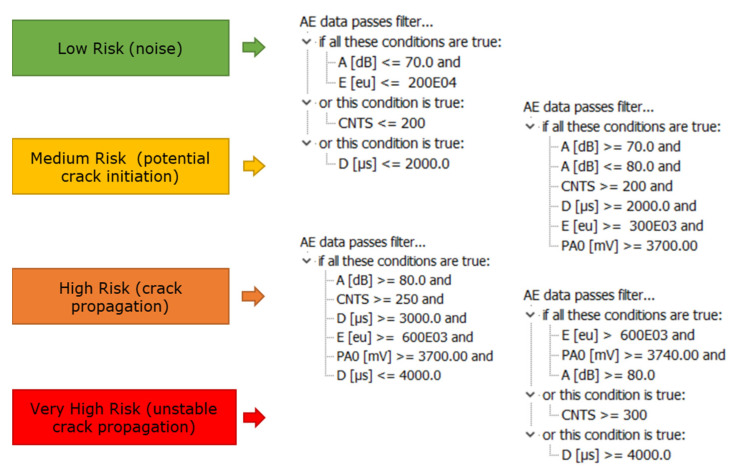
Filtering criteria established per category.

Taking into account this behaviour, warning criteria can be defined for the real-time SHM of a mooring chain under fatigue loading, based on the present experimental conditions. This criteria and subsequent alarm setting will be based on the researched characteristics for crack growth identification and will consist of the following factors (Figure 15).

Depending on the environmental conditions and noise, if medium risk is determined, there will be an indication of crack initiation but it would be difficult to conclude the extent of the damage. 

If characterised as high risk, the burst will be recorded as a potential crack propagation occurrence and the evolution will be closely followed and monitored. In addition, if either counts > 300 or duration > 4000 µs, the hit would be characterised as critical, and indicate prospective high risk due to the potential presence of unstable crack growth. 

**Figure 16:**
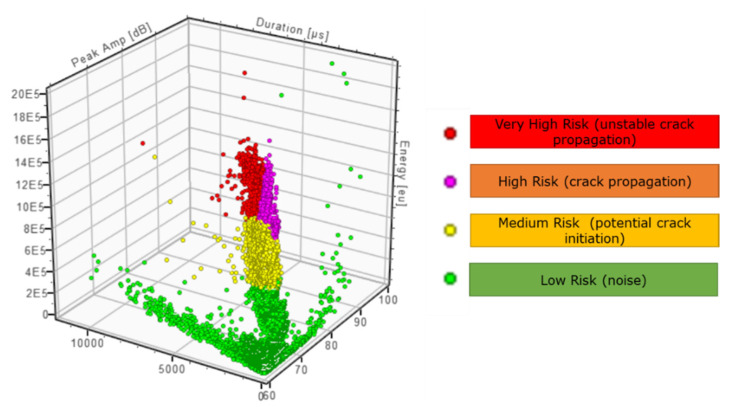
Data classification based on low, medium, high, and very high risk.

The outcome of the data clustering for the current experiment based on the defined filtering criteria can be observed in Figure 16. These four clusters correspond to data considered within the four categories: low, medium, high, and very high risk. This pre-programmed clustering will allow real-time visualisation of the damage evolution and the risk, which is ultimately the digested information that is required as part of SHM plans. 

In addition to the alarm-setting tool, a procedure must be defined in order to establish a plan of action based on the asset’s operational parameters, location, associated risk, asset criticality, and other factors.

## 6. Conclusions 

The work presented in this paper included the use of theoretical, operational, and signal-related data to provide a common method to analyse crack initiation and growth based on the results of a large-scale chain fatigue test rig. Different signal parametric trends and consequent plots with distinct variables were sampled and analysed to determine their added value during the assessment of the data feeding into fatigue crack growth evaluation. 

In summary, for the data analysis, amplitudes in the range of 80–85 dB can be taken as indications of crack growth, while crack initiation would occur in the region of 70–80 dB. Similarly, absolute energy exceeding 6 × 10^5^ eu can be used to identify crack growth-related events. Environmental noise would exhibit low amplitudes, not exceeding 70 dB, and absolute energies would not exceed 3 × 10^5^ eu. An increase in the average frequency was also observed as the crack growth increased in size and propagation speed. 

**Figure 17:**
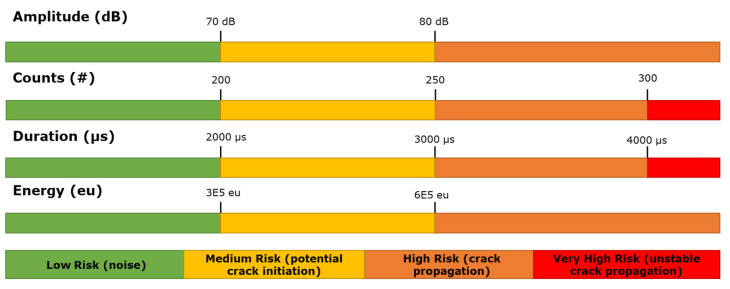
Filtering criteria and risk level.

Filtering and clustering criteria have been defined to successfully characterise four different clusters of events, three of which are directly related to the cracking phases: initiation, growth, and final growth. Based on this, a colour-coded alarm for risk identification can provide warnings based on low, medium, high, and very high risk stages. The analysis and definition of limits of the parameters, and the correlation of their characteristics with the crack, helped to identify filtering approaches to discriminate between noise, initiation, and growth-related events. Based on these, the filtering criteria shown in Figure 17 were established, to support the identification of different crack growth stages and noise. 

Environmental noise must be explored based on each scenario, in order to reinforce the ability to extract information from signals so that the important features become easily discernible. The application of any noise reduction method, usually a filter, has to be preceded by a clear knowledge of which aspects of the signals need to be retained (e.g., linear, statistical, time-scale filters). 

A disadvantage of AE is that systems can normally qualitatively estimate the extent of damage or size of the defect. Monitoring methods are set in place to reinforce the wider structural integrity strategies, and other NDT methods are still needed for a more exhaustive examination and to provide quantitative results. Conventional ultrasonic evaluation is often used to assess AE indications. Another disadvantage is that AE can be sensitive to environmental noise, making it difficult to separate material emissions from the noise made by the operational environment. This can be a limitation if adequate noise identification and reduction strategies are not considered. 

The optimum AE parameters must be estimated for different applications. The appropriate selection and installation of the AE sensors are crucial for a precise data collection strategy. The data must be processed to determine crack initiation and growth and to discriminate irrelevant information. 

The ultimate aim of SHM and SI strategies is not only to detect faults but also to provide diagnosis and prognosis to predict damage growth. This investigation has focused on the detection and sizing of flaws. Further work must be carried out for modelling prognostics of fatigue crack growth to reinforce predictive maintenance plans.

## Figures and Tables

**Table 1:** Calibration and signal reproducibility verification plan.

Pencil Lead Break	Sensor Pulsing
A PLB simulates AE events PLBs at ±10 cm (4 at −10 cm, 4 at +10 cm) PLBs at link inner face opposing weld (4 PLBs per link) Transmission across links was observed Amplitude dropped from 85–90 dB in the first link to ~65 dB after transmission through two consecutive sensors (average) PLB in air and underwater	Emission of ultrasound pulses by the sensor Pulsing from the sensor, four pulses per sensor Transmission across links was observed Amplitude dropped from 80 dB in the first link to ~70 dB after transmission through two consecutive sensors (average) Pulsing in air and underwater

**Table 2:** Data monitoring periods and data structure.

ID	File Name	Size and Duration	State	Period Notes
P1	Period 1	7 GB (25 × 104 s)	No crack	Data monitoring starts
P2	Period 2	2.7 GB (16 × 10^4^ s)	Crack initiation	Period leading to C1
C1	Crack 1—MPI 5 mm			
P3	Period 3	2.8 GB (6 × 10^5^ s)	Crack growth	C1 developing into C2
P4	Period 4	3.2 GB (5 × 10^5^ s)	Crack growth end	Period leading to C2
C2	Crack 2—MPI 120 mm crack			
END	End of the test			

**Table 3:** Parametrical filtering criteria based on cracking stage.

Parameter	Noise	Initiation	Growth	Unstable Growth
Amplitude (dB)	A < 70	70 < A < 80	A > 80	A > 80
Counts (#)	C < 200	200 < C < 250	250 < C < 300	C > 300
Duration (µs)	D < 2000	2000 < D < 3000	3000 < D < 4000	D > 4000
Energy (eu)	E < 3 × 10^5^	3 × 10^5^ < E < 6 × 10^5^	6 × 10^5^ < E < 22 × 10^5^	6 × 10^5^ < E < 22 × 10^5^
Average Frequency (kHz)	1 < AF < 130	25 < AF < 120	70 < AF < 110	75 < AF < 100

## Data Availability

Not applicable.
